# Elevated RACGAP1 Expression Enhances Malignant Potential in Lung Adenocarcinoma and Serves as a Prognostic Factor

**DOI:** 10.7150/jca.96334

**Published:** 2024-06-03

**Authors:** Wei Lai, Yunshu Su, Yangbo Li, Yifan Zuo, Kunzhuo He, Tianyu Zhang, Dunyu Peng, Wei Wang

**Affiliations:** 1 Department of Thoracic Surgery, Renmin Hospital of Wuhan University, Wuhan 430060, China.; 2 Department of anesthesiology, Renmin Hospital of Wuhan University, Wuhan 430060, China.

**Keywords:** RACGAP1, lung adenocarcinoma, prognostic model, invasion

## Abstract

**Background:** While RACGAP1 is identified as a potential oncogene, its specific role in lung adenocarcinoma (LUAD) remains unclear.

**Methods:** First, we conducted a comprehensive analysis of the role of RACGAP1 across 33 types of cancer. Subsequently, we investigated the expression levels of RACGAP1 and its impact on prognosis using data from The Cancer Genome Atlas (TCGA) database. We utilized single-cell sequencing data to explore the tumor-related processes of RACGAP1 in LUAD and validated our findings through experimental verification. Employing a consensus clustering (CC) approach, we subdivided LUAD patients into two subtypes based on RACGAP1 cell cycle-related genes (RrCCGs). These subtypes exhibited significant differences in tumor characteristics, lymph node metastasis, and recurrence. Furthermore, we evaluated the prognostic influence of RrCCGs using univariate Cox regression and least absolute shrinkage and selection operator regression models (LASSO), successfully establishing a prognostic model.

**Results:** RACGAP1 is frequently overexpressed in various tumors and can impact the prognosis of patients with LUAD. Additionally, experimental evidence has demonstrated that low expression of RACGAP1 favors tumor cell apoptosis and restoration of the cell cycle, while high expression promotes invasion and metastasis. Through CC analysis of RrCCGs, patients were classified into two groups, with survival analysis revealing distinct prognoses and stages between the two groups. Furthermore, Cox and LASSO regression successfully constructed a prognostic model with robust predictive capability.

## Introduction

Lung cancer ranks among the most lethal malignancies globally, with lung adenocarcinoma (LUAD) representing a staggering 50% of all lung cancer 1. LUAD exhibits a marked propensity for metastasis and recurrence, often showcasing resistance to conventional chemotherapy regimens 2. Despite recent strides in LUAD treatment, long-term survival rates remain dismally low. Mounting evidence underscores the superiority of multi-targeted therapeutic approaches over single-agent interventions across diverse cancer types. Hence, the identification of more dependable drug targets assumes paramount importance.

RAC GTPase-activating protein 1 (RACGAP1), also known as CDAN3B or CYK4, is a member of the GTPase-activating protein family 3. In recent years, it has been found that RACGAP1, as a newly discovered oncogene, plays an important role in intracellular signaling pathway transduction by regulating the activity of Rho family GTPase proteins, such as cytokinesis cycle protein 42 and RAC1, which promotes the conversion of GTP hydrolysis to GDP 4. Its role in the process of tumor development has gradually received attention, and it has been found that RACGAP1 is involved in the development of a variety of tumors. Early clinical studies have indicated a close association between RACGAP1 expression and patient prognosis 5. In recent years, mounting evidence has underscored the significant role of RACGAP1 across various malignancies. For instance, it has been elucidated that RACGAP1 can enhance the aggressiveness of tumors and facilitate lymph node metastasis in colorectal cancer 6. Additionally, in hepatocellular carcinoma, RACGAP1 interacts with HIF-1alpha, thereby exerting influence on hepatocarcinogenesis 7. Moreover, RACGAP1 has emerged as a pivotal player in cervical cancer 8. In esophageal and nasopharyngeal carcinomas, RACGAP1 also exhibits pro-carcinogenic effects 9, 10. However, its role in other types of cancers is still unknown, especially in the field of LUAD. Due to the progress in next-generation sequencing technologies and the accessibility of datasets like The Cancer Genome Atlas (TCGA) dataset, researchers are able to gain open access to genomic and transcriptomic data of common cancers, which provides essential support for analyzing and revealing the predictive value of RACGAP1 in cancer prognosis and precision medicine.

Our study first explored the role of RACGAP in 33 cancers. In addition, by examining cancer-related cellular behaviors using the Cancer Single-cell State Atlas (CancerSEA) database, we found that RACGAP1 was closely associated with cell cycle, epithelial-mesenchymal transition (EMT) and invasion. Subsequent experimental validation solidified these findings. We used the Least absolute shrinkage and selection operator regression algorithm (LASSO) to identify prognostic genes of cell cycle genes associated with RACGAP1 in LUAD. We analyzed the related genes by consensus clustering and classified them into two subtypes. Each subtype exhibited different clinical, molecular and cellular features. Finally, we constructed a prognostic model based on the prognostic genes.

## Materials and methods

### Data acquisition and gene expression analysis

We downloaded RNA sequencing and clinical data for LUAD from the TCGA database (https: //www.cancer.gov/ccg/research/genome-sequencing/tcga). The TCGA data were utilized to construct the cohort for subsequent prognostic model development. The gene expression matrix included 59429 genes and 557 samples. Gene-related annotations were performed using Genocode (GENCODE.v32) (ftp://ftp.ebi.ac.uk/pub/databases/gencode/Gencode_human/). We analyzed the expression of RACGAP1 in 33 cancers in TCGA using the online database Timer1.0 (https://cistrome.shinyapps.io/timer/). For some cancers lacking normal or tumor samples (e.g., TCGA- OV and TCGA-LAML), we used the Gene Expression Profiling Interactive Analysis (GEPIA) (https://gepia.cancer-pku.cn) to compare RACGAP1 expression in tumor tissues with normal tissues 11. This site integrates tumor data from TCGA with gene expression data from various normal tissues from the organization, including the Genotypic Tissue Expression (GTEx) database (https://www.genome.gov/Funded-Programs-Projects/Genotype-Tissue-Expression-Project). In addition, the expression levels of RACGAP1 in all TCGA tumors at different stages were shown using the GEPIA database. Subsequently, we explored the expression levels of RACGAP1 in normal and tumor tissues using the UALCAN database (https://ualcan.path.uab.edu/analysis-prot.html), which collects data from the Clinical Proteomics Tumor Analysis Consortium and includes expression information for approximately 10,000 proteins. Immunohistochemical data were obtained using the HPA database (https://www.proteinatlas.org/).

### Correlation analysis and discovery of RACGAP1-related cell cycle genes

We used the CancerSEA (http://biocc.hrbmu.edu.cn/CancerSEA/) database to explore the relationship between RACGAP1 and 14 primary tumor-associated cellular activities (e.g., cell cycle, EMT and invasion) 12. Correlations between RACGAP1 and cellular activity scores were plotted using the R package "ggplot2", and heatmaps were created using the "heatmap" tool. Then, we identified genes associated with RACGAP1 expression in LUAD by Pearson correlation analysis (*P*<0.05, | r |>0.6) (see **additional file 1** for details). Subsequently, we downloaded 967 cell cycle-related genes from the CancerSEA database and used the "VennDiagram" package to create a Venn diagram, which was considered RACGAP1-related cell cycle genes (RrCCGs).

### Subtype discovery of RACGAP1-related cell cycle genes

We utilized the “ConsensusClusterPlus” tool to conduct a clustering analysis on RrCCGs, aiming to delineate intrinsic groups sharing similar biometric characteristics 13. This analysis involved 1000 bootstraps, with each subsample comprising 80% of the patient cohort, while setting the clustering range from 2 to 9. We discerned the optimal partitioning scheme by assessing the consensus matrix and consensus clustering (CC) distribution function.

### Prognostic signature development and evaluation for LUAD

The RrCCGs were partitioned into distinct training and validation sets. Subsequently, a prediction signature was formulated based on the training set, with its predictive efficacy validated in the independent validation set. Initially, univariate Cox proportional hazards regression analysis was conducted to evaluate the association between RrCCGs and overall survival (OS) within the training set (*P* < 0.05). To mitigate overfitting, the Least Absolute Shrinkage and Selection Operator (LASSO) technique, implemented through the R package 'glmnet', was employed to penalize the Cox proportional hazards regression, thereby reducing the number of genes included in the model and curtailing complexity. Stepwise Cox regression analyses utilizing the Akaike Information Criterion (AIC) were then employed to identify the optimal gene subset for constructing risk models aimed at prognosticating LUAD outcomes. The risk score formula was defined as follows: risk score = (expression level of gene a * coefficient a) + (expression level of gene b * coefficient b) + ... + (expression level of gene n * coefficient n).

The clinical information of patients from TCGA was utilized to construct prognostic models. Patients were then stratified into high-risk and low-risk cohorts according to the median risk score. Survival curves for RrCCGs were plotted to illustrate the survival disparities between patients in the high and low-risk categories. Moreover, multifactorial score plots, also known as nomograms, were constructed by combining clinical information (age, gender, stage, TNM stage) and risk scores, enabling clinicians to quickly and accurately predict the survival of LUAD patients.

### Cell culture, reagents, and transfection

A549 and H1299 cell lines were procured from the American Type Culture Collection (ATCC, USA) and maintained under standard conditions. A549 cells were cultured in F-12k medium supplemented with 10% fetal bovine serum (FBS), 50 mg/mL streptomycin, and 50 IU/mL penicillin, all sourced from Pricella (Wuhan). H1299 cells were cultured in RPMI 1640 medium supplemented with 10% FBS, 50 mg/mL streptomycin, and 50 IU/mL penicillin. Cultures were maintained at 37°C in a humidified atmosphere with 5% CO2. Transfection of small interfering RNA (siRNA) was facilitated using Lipofectamine 8000 (Beyotime, Shanghai). The specific siRNA sequences utilized were as follows: S1: 5'-GCUGAAGCAUGCACGUAAUTT-3', AS1: 5'-AUUACGUGCAUGCUUCAGCTT-3'; S2: 5'-CCCUGGACCUGUAAAGAAA-3', AS2: 5'-GCUGAAGCAUGCACGUAAU-3'. Post-transfection, cells were incubated for 6 hours before refreshing the culture medium.

### Wound healing assay

A549 and H1299 cells were seeded onto six-well plates (Servicebio, Wuhan) and allowed to reach approximately 90% confluency. Subsequently, wounds were generated using a sterile 200 μl pipette tip. The wounded cells were then washed thrice with phosphate-buffered saline (PBS) sourced from Servicebio (Wuhan, China) to eliminate cellular debris. Following this, cells were incubated in fresh medium for 0 and 24 hours to monitor wound closure. The percent wound closure was determined using the formula: Percent wound closure (%) = (initial wound area - final wound area)/initial wound area.

### Cell viability assay (CCK8)

A549 and H1299 cells were seeded into separate 96-well plates (Servicebio, Wuhan), with each well containing 100 μl of medium and a cell density of 1000 cells per well. Following 24 hours of incubation, transfection was carried out, with each experimental group having five replicates. At 0 hours, 24 hours, and 48 hours post-transfection, 10 μl of CCK8 reagent (Glpbio, America) was added to each well. Subsequently, absorbance readings at 450 nm were recorded to assess cell viability and proliferation.

### Colony formation assay

Logarithmic phase A549 and H1299 cell suspensions were harvested, and 3000 cells per well were seeded into 6-well plates. Following a 14-day incubation period, colony formation was terminated. Colonies were fixed using 4% paraformaldehyde obtained from Servicebio (Wuhan), followed by staining with 0.5% crystal violet (Servicebio, Wuhan). Subsequently, the results of colony formation were documented, and images were captured to document the technical replicates of the clones.

### Transwell assay

Cell migration and invasion assays were conducted utilizing Transwell chambers (Corning, America). For migration assays, cells in the logarithmic growth phase were harvested and suspended in serum-free medium, with a cell density adjusted to 2.5 × 10^5 cells/mL. Subsequently, 200 μl of cell suspension was added to the upper chamber, while 500 μl of medium containing 10% FBS was added to the lower chamber. Following a 48-hour incubation period, cells were fixed with 4% paraformaldehyde for 30 minutes and then stained with a 0.5% crystal violet solution for 10 minutes. Afterward, non-migrated cells on the upper side of the membrane were gently removed with a cotton swab, and the migrated cells on the lower side were observed and counted in 5 randomly selected fields under a 400x microscope (Olympus, Japan).

For invasion assays, prior to seeding cells, it was necessary to thaw Matrigel (Corning, America) and add it to the upper chamber. The Matrigel-coated upper chamber was then incubated in a cell incubator for 2 hours to allow the Matrigel to hydrate. The subsequent steps were identical to those of the migration assays.

### Western blot

The cells were lysed on ice, and total protein was extracted using RIPA buffer (Servicebio, Wuhan). Subsequently, protein samples were separated by 10-12% SDS polyacrylamide gradient gel electrophoresis and transferred onto a PVDF membrane. To minimize non-specific binding, the membrane was swiftly incubated with Rapid Closure Solution (Epizyme, Shanghai) for 30 minutes. Primary antibodies were then applied and incubated at room temperature for 1-2 hours, followed by secondary antibodies of either mouse or rabbit origin. Protein signals were detected using the BioRad ChemiDoc XRS+ system or the Monad QuickChemi 5200. GAPDH served as an internal reference for normalization. The relevant antibodies, including E-carderin (20874-1-AP), N-carderin (66219-1-AP), Vimentin (660330-1-lg), and GAPDH (60004-1-lg), were procured from Proteintech (Wuhan). The RACGAP1 antibody (ES3299) was obtained from ELK Biotechnology (Wuhan).

### Immunofluorescence staining

A549 and H1299 cells (50,000 cells per well) were seeded into 6-well culture plates. Twenty-four hours post-transfection, cells were fixed with 4% paraformaldehyde for 10 minutes at room temperature. Following fixation, cells were washed three times with PBS. Subsequently, 200 μl of appropriately diluted antibodies, specifically Cohesion bioscience's CRG1033 for A549 cells and CRG1035 for H1299 cells, were added to each well according to the manufacturer's instructions. After a 30-minute incubation period at room temperature, cells were washed three times with PBS. Next, the cells were stained with DAPI stain for 3 minutes at 25°C to visualize nuclear DNA, and fluorescence signals were observed under a fluorescence microscope (Olympus, Japan).

### Apoptosis detection

After detaching the cells with trypsin treatment (without EDTA), the cell suspension was collected, and three washes were conducted using pre-cooled PBS. Under conditions avoiding light exposure, Annexin V-FITC (Elabscience, Wuhan) and propidium iodide (PI, Elabscience, Wuhan) fluorescent dyes were added to the cell suspension and incubated for 30 minutes at room temperature. Subsequently, the fluorescence intensity of each channel was measured using flow cytometry equipment (from Beckman Coulter, America).

### Cell cycle detector

Cells were digested using EDTA-free trypsin, followed by fixation treatment with pre-cooled 75% ethanol and left to stand overnight at 4°C. Afterwards, three PBS washes were performed and 10 µl of RNA, 200 PI and 200 µl of PBS were added to the samples according to the guidelines of the product manual. Finally, the fluorescence intensity of each channel was detected using flow cytometry.

### Statistical analysis

The data were presented as mean ± standard deviation (mean ± SD). Statistical analysis was performed using SPSS 25.0, and graphical representations were by using GraphPad Prism 9.5.0 software. The t-test was employed to assess differences between two groups, while one-way analysis of variance (ANOVA) followed by Sidak's and Tukey's tests were used to evaluate between-group differences. Statistical significance was set at *P* < 0.05. Other analyses were performed in R software (version 4.2.1).

## Results

### RACGAP1 expression analysis in 33 cancer tumors

**Figure 1:**
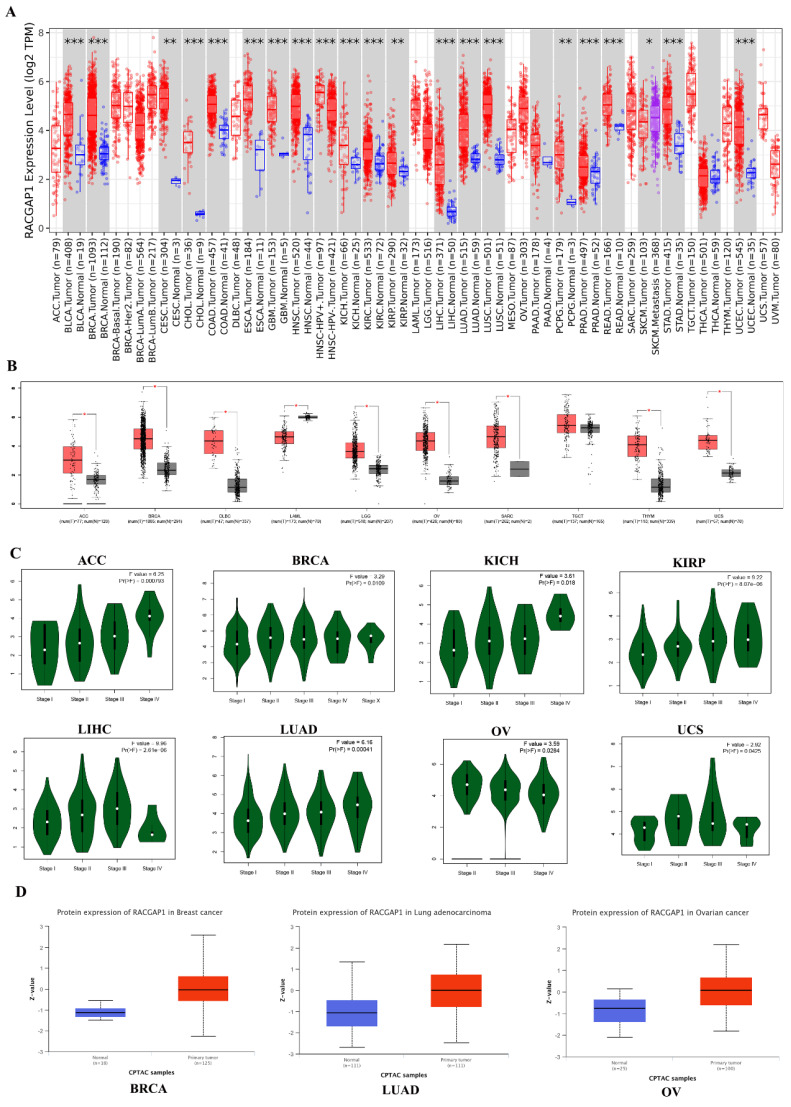
** Pan-cancer analysis of RACGAP1. A** The expression pattern of RACGAP1 across 33 cancer types was analyzed using the TIMER database (https://cistrome.shinyapps.io/timer/). **B** For samples lacking normal or tumor tissue, GETx data were supplemented and analyzed using the GEPIA online tool (https://gepia.cancer-pku.cn). **C** The expression pattern of RACGAP1 across different stages of tumors was analyzed using GEPIA (only showing significant differences). **D** The expression levels of RACGAP1 in normal and tumor tissues of BRCA, LUAD, and OV were analyzed using the UALCAN database (https://ualcan.path.uab.edu/analysis-prot.html). (*P<0.05, **P<0.01, *** *P*<0.001).

We analyzed RACGAP1 expression across 33 cancer types using the Timer1.0 database (https://cistrome.shinyapps.io/timer/) (**Figure 1 A**). Given the absence of available normal or tumor tissue samples, we complemented our investigation of RACGAP1 expression using the online tool GEPIA (https://gepia.cancer-pku.cn), which incorporates gene expression data from the GTEx project (https://www.genome.gov/Funded-Programs-Projects/Genotype-Tissue-Expression-Project) for both normal and tumor tissues (**Figure 1 B**). Our findings indicated a general upregulation of RACGAP1 expression across most tumor types. Furthermore, GEPIA provided additional insights into RACGAP1 expression across different stages of adrenocortical carcinoma (ACC), bladder invasive carcinoma (BRCA), kidney chromophobe (KICH), kidney renal papillary cell carcinoma (KIRP), liver hepatocellular carcinoma (LIHC), LUAD, ovarian serous cystadenocarcinoma (OV), and uterine carcinosarcoma (UCS) (**Figure 1 C**). Subsequently, we utilized the UALCAN online tool (https://ualcan.path.uab.edu/analysis-prot.html) to scrutinize discrepancies in protein expression levels between normal and tumor tissues in BRCA, LUAD, OV, and LIHC (**Figure 1 D and Supplementary Figure 1**).

### Regulation of Cancer-Associated Cellular Processes (LUAD) by RACGAP1

**Figure 2:**
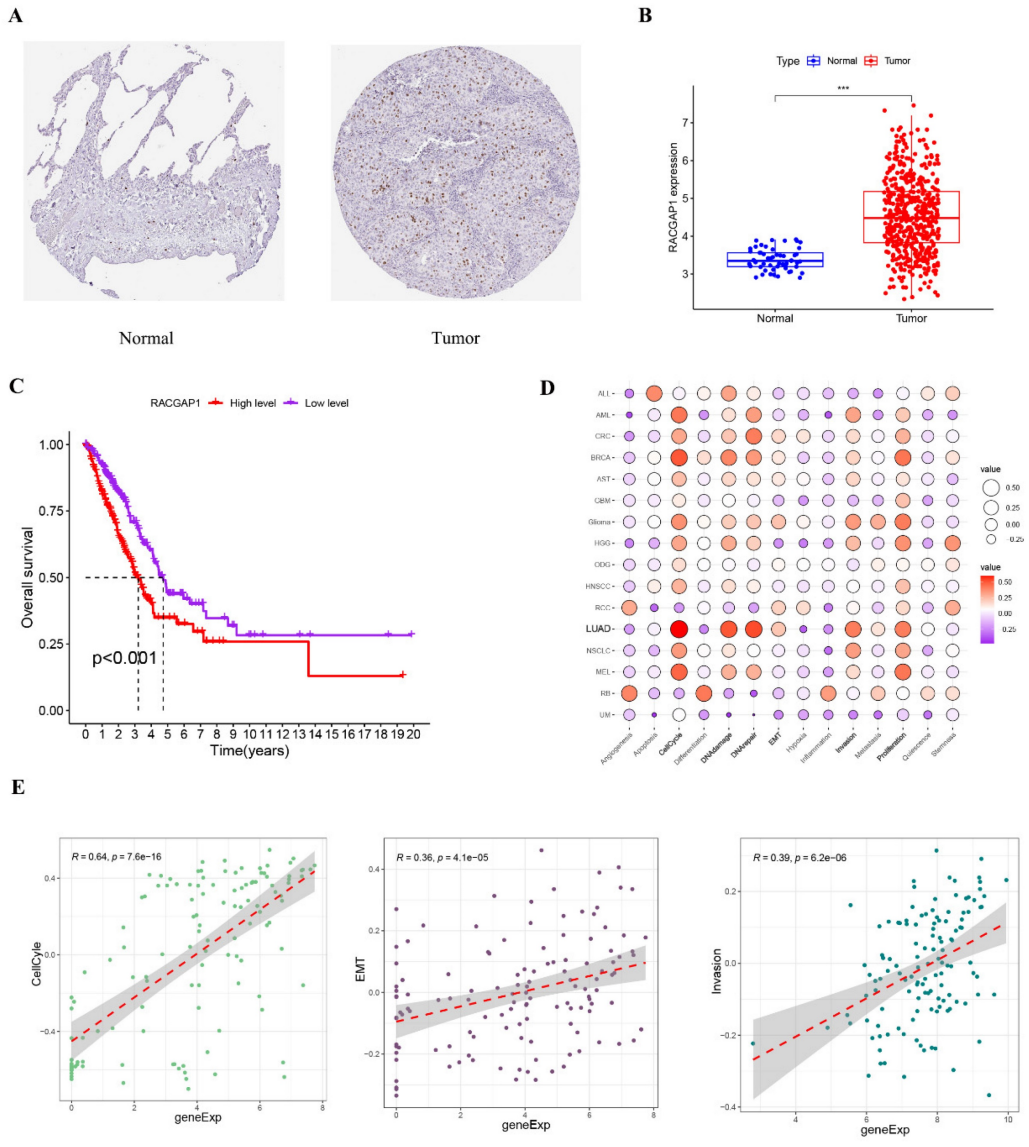
Expression of RACGAP1 in LUAD and its involvement in cancer-related cellular processes. **A.** Immunohistochemistry images depicting the expression of RACGAP1 in normal and tumor tissues, sourced from HPA database (https://www.proteinatlas.org/). **B.** Expression profiles of RACGAP1 in normal and tumor tissues sourced from TCGA. **C.** Clinical data from TCGA illustrating the survival outcomes associated with high and low expression levels of RACGAP1.** D.** Association between the RACGAP1 gene and 14 functional states across 33 types of cancer, with red indicating positive correlation and purple indicating negative correlation, analyzed using the online platform CancerSEA (http://biocc.hrbmu.edu.cn/CancerSEA/). **E.** Correlation between RACGAP1 and cellular processes including cell cycle, invasion, and EMT. (*P<0.05, **P<0.01, *** *P*<0.001).

We first obtained immunohistochemical images of LUAD from the HPA database and found that RACGAP1 expression was elevated in tumor tissues (**Figure 2 A**). Subsequently, we used the RACGAP1 expression data from the TCGA database and the clinical data to find that the expression level of RACGAP1 was elevated in tumor tissues, which was consistent with the immunohistochemical results from the HPA database (**Figure 2 B**). Clinical data showed that high expression of RACGAP1 was associated with shorter survival (**Figure 2 C**). To deepen our understanding of the relationship between RACGAP1 and cancer-related cellular processes, we subjected it to analysis using the CancerSEA database. The results underscored RACGAP1's significant associations with pivotal processes such as the cell cycle, DNA damage, repair, cell invasion and proliferation (**Figure 2 D and Supplementary Figure 2**). Furthermore, **Figure 2 E** illustrates the correlation between RACGAP1 and key cellular processes including the cell cycle, EMT, and invasion.

### Correlation between RACGAP1 and cell proliferation and invasion

**Figure 3:**
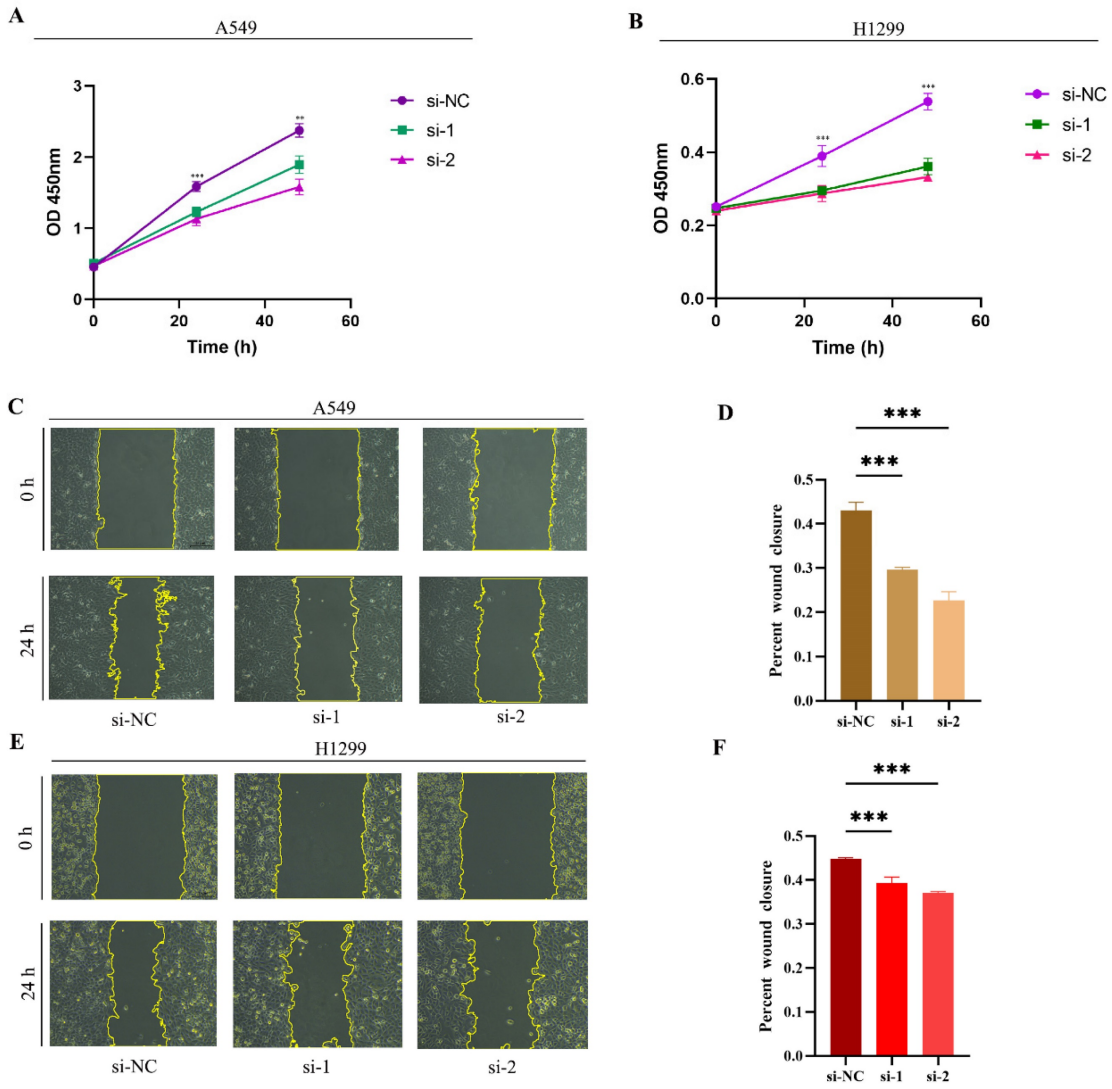
** Effects of RACGAP1 Silencing on Cell Viability and Proliferation**. **A:** CCK-8 assay demonstrates a significant decrease in growth rate of RACGAP1-silenced A549 cells over time. **B:** CCK-8 assay indicates a marked reduction in growth rate of RACGAP1-silenced H1299 cells over time. **C-D:** Wound healing assay data reveal decreased migratory ability of A549 cells following RACGAP1 silencing (Scar bar = 25μm). **E-F:** Wound healing assay data show reduced migratory capacity of H1299 cells after RACGAP1 silencing (Scar bar = 25μm). (*P<0.05, **P<0.01, *** *P*<0.001).

We conducted experiments using two classical LUAD cell lines, A549 and H1299. Initially, we investigated the impact of siRNA-mediated RACGAP1 gene silencing on cell viability. In A549 cells, siRNA-mediated RACGAP1 silencing resulted in significant changes in cell viability at both 24- and 48 hours post-treatment (**Figure 3 A**). Similarly, in H1299 cells, cell viability showed a notable decrease following the same silencing treatment (**Figure 3 B**). Subsequently, we conducted a wound healing assay, demonstrating a significant reduction in cell proliferation and migration following RACGAP1 silencing (**Figure 3 C-F**).

**Figure 4:**
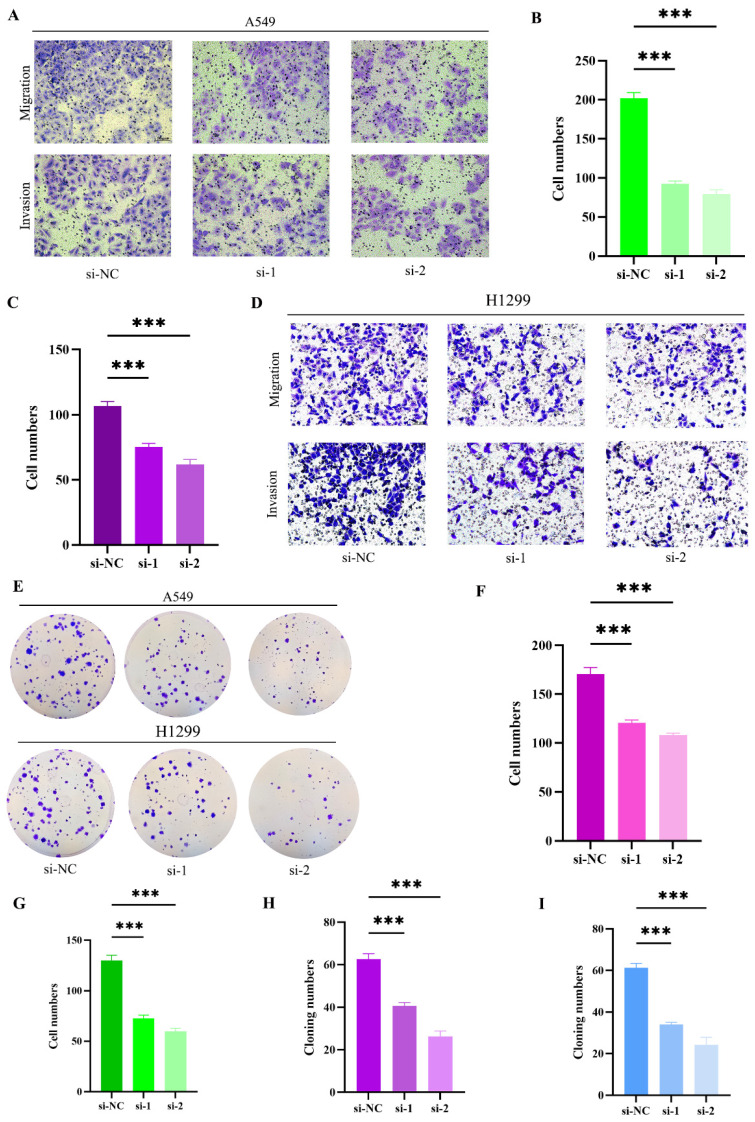
** RACGAP1 Silencing Suppresses Tumor Cell Proliferation, Migration, and Invasion. A-C:** Effects of RACGAP1 silencing on migration and invasion of A549 cells (Scar bar = 50μm). **B:** Quantitative data for A549 cell migration; **C:** Quantitative data for H1299 cell invasion. **D:** Impact of RACGAP1 silencing on H1299 cells (Scar bar = 50μm). **E:** Congenic assay assessing the effect of RACGAP1 on tumor cell proliferation. **F:** Quantitative analysis of H1299 cell migration; **G:** Quantitative analysis of H1299 cell invasion; H: Quantitative assessment of A549 colony formation; **I:** Quantitative assessment of H1299 colony formation. (*P<0.05, **P<0.01, *** *P*<0.001).

To further elucidate the impact of RACGAP1 silencing on cell migration, invasion, and proliferation, we employed transwell chambers for migration and invasion assays. Initially, siRNA transfection was conducted on A549 cells, revealing a significant reduction in migrating and invading cells post-RACGAP1 silencing (**Figure 4 A-C**). This effect was similarly observed in H1299 cells (**Figure 4 D, F-G**). To corroborate the influence of RACGAP1 on the proliferative capacity of LUAD cells, we conducted cell cloning experiments, demonstrating a notable decrease in the number of clones following RACGAP1 silencing (**Figure 4 E, H-I**). In conclusion, our findings indicate that RACGAP1 plays a pivotal role in promoting the proliferation, migration, and invasion of tumor cells, and its reduced expression significantly inhibits these effects.

### The relationship between RACGAP1 and apoptosis and cell cycle regulation

**Figure 5:**
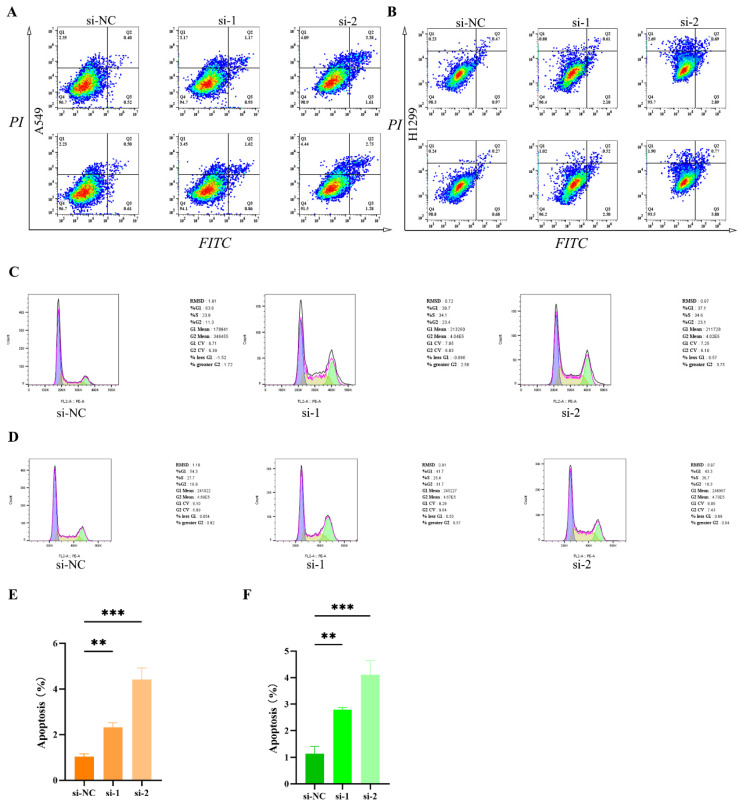
**Increased tumor cell apoptosis and cell cycle arrest following RACGAP1 silencing. A** The impact of RACGAP1 on apoptosis in A549 cells. **B** The effect of RACGAP1 on apoptosis in H1299 cells. **C** RACGAP1's influence on the cell cycle in A549 cells. **D** The effect of RACGAP1 on the cell cycle in H1299 cells. **E** Quantitative analysis of apoptosis in A549 cells. **F** Quantitative analysis of apoptosis in H1299 cells. (*P<0.05, **P<0.01, *** *P*<0.001).

To further elucidate the impact of RACGAP1 on apoptosis and cell cycle progression, we conducted comparative analyses of apoptotic cell counts pre- and post-silencing of RACGAP1 in A549 and H1299 cell lines. Our findings reveal a significant increase in apoptotic cell populations upon RACGAP1 silencing in both A549 (**Figure 5 A, E**) and H1299 cell lines (**Figure 5 B, F**). Additionally, we utilized PI staining to investigate cell cycle dynamics and observed a pronounced cell cycle arrest in RACGAP1-silenced A549 cells (**Figure 5 C**), mirroring similar observations in H1299 cells (**Figure 5 D**). Thus, our results indicate that RACGAP1 silencing promotes apoptosis and induces cell cycle arrest in LUAD, underscoring its potential as a therapeutic target.

### The relationship between RACGAP1 and Epithelial-Mesenchymal Transition (EMT)

**Figure 6:**
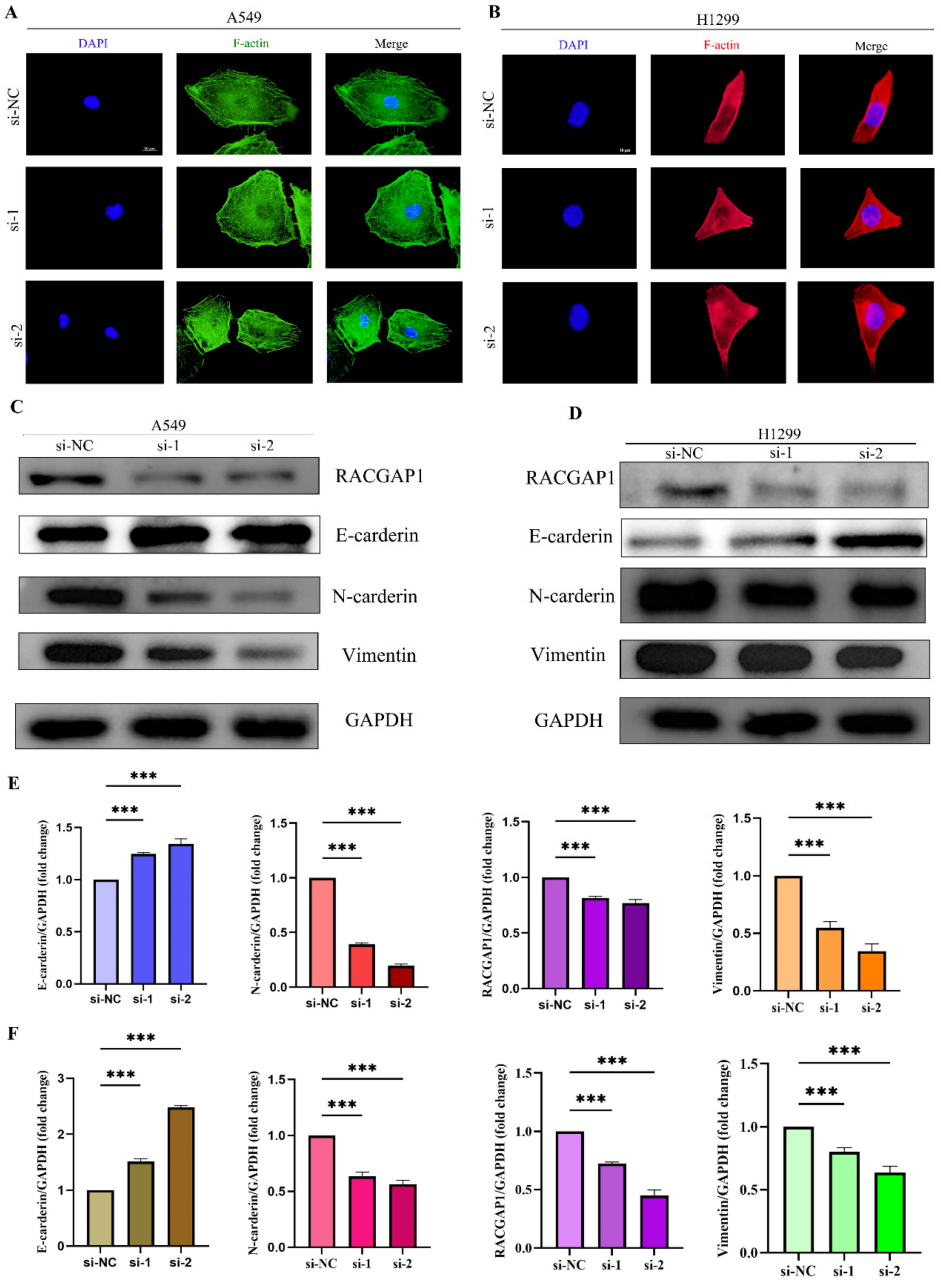
** Immunofluorescence and Western Blot Analysis of RACGAP1's Impact on Epithelial-Mesenchymal Transition. A** Immunofluorescence staining of A549 cells with F-actin. **B** Immunofluorescence staining of H1299 cells with F-actin. **C** Western blot analysis of epithelial-mesenchymal transition marker protein changes in A549 cells before and after knockdown.** D** Western blot analysis of epithelial-mesenchymal transition marker protein changes in H1299 cells before and after knockdown. **E** Quantitative protein analysis in A549 cells. F) Quantitative protein analysis in H1299 cells. (*P<0.05, **P<0.01, *** P<0.001).

To delve deeper into the influence of RACGAP1 on EMT, we initially employed immunofluorescence techniques to visualize F-actin protein in A549 and H1299 cells (**Figure 6 A-B**). Upon RACGAP1 knockdown, we observed significant alterations in cell morphology, indicative of an epithelial-like transformation. To reinforce this observation, we conducted western blot analysis, which unveiled an upsurge in epithelial markers alongside a decline in mesenchymal markers within the knockdown group (**Figure 6 C-F**). Consequently, our findings suggest that heightened expression of RACGAP1 facilitates the EMT process in tumor cells, elucidating its potential role in cancer metastasis.

### Refinement and assessment of LUAD subgroups through consensus clustering

**Figure 7:**
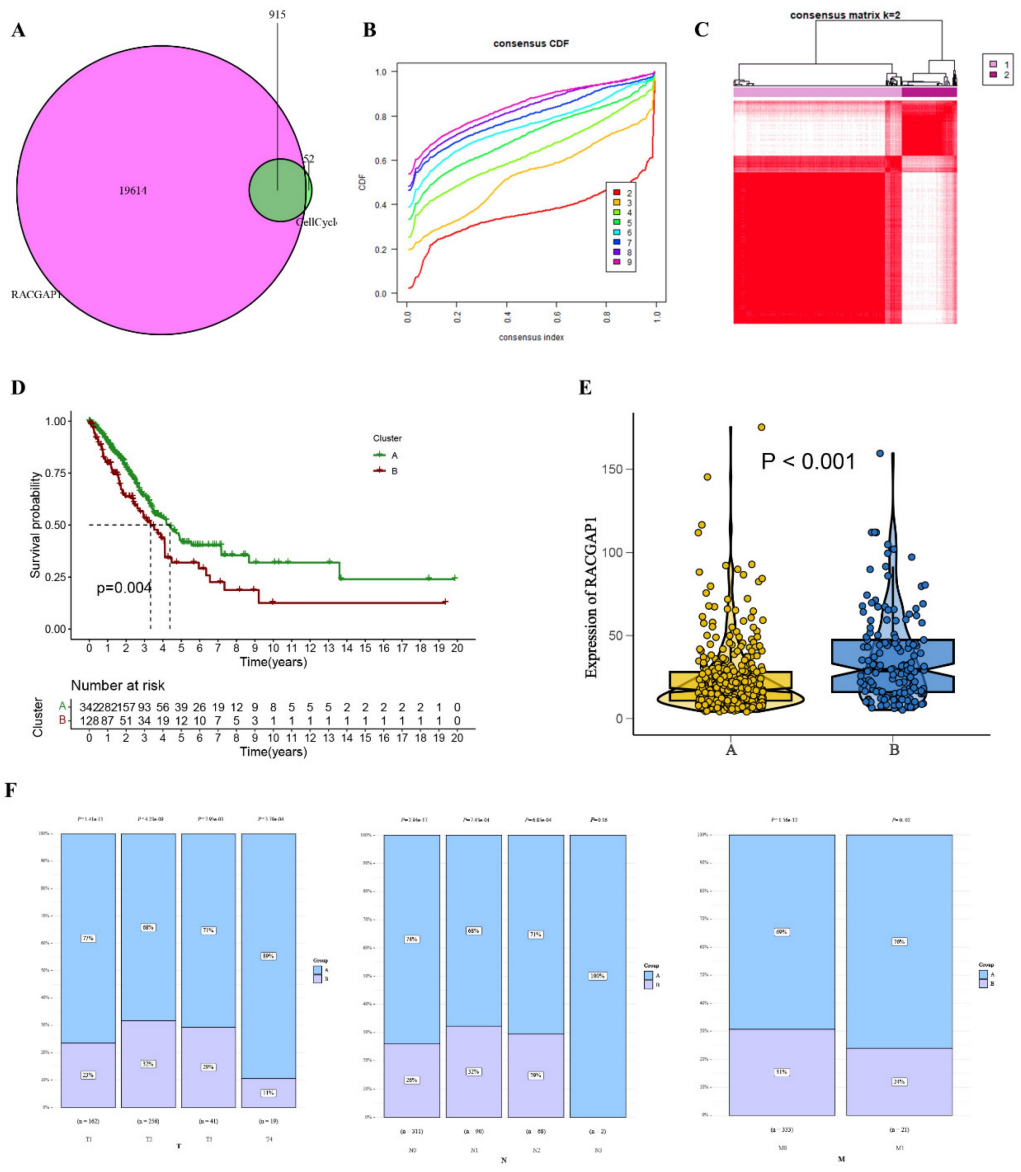
Clustering analysis of the cell cycle-related gene RACGAP1 **A.** Venn diagram displaying common genes between RACGAP1-related genes and cell cycle genes; **B.** Cumulative distribution function; **C.** Heatmap illustrating sample clustering of RrCCGs; **D.** Survival curves of clustering; **E.** Expression patterns of RrCCGs in different clusters;** F.** Distribution of TNM staging under different clusters. (*P<0.05, **P<0.01, *** *P*<0.001).

In our comprehensive analysis of TCGA, we scrutinized 915 genes closely linked with the cell cycle, as illustrated in **Figure 7 A and additional file 1.** Employing cumulative distribution functions, we observed that when selecting K=2, the RrCCGs exhibited notable clustering tendencies, as depicted in **Figure 7 B-C.** Notably, survival curves revealed that patients categorized in cluster A exhibited a notably improved prognosis relative to their counterparts in cluster B (**Figure 7 D**). Further exploration within cluster A unveiled diminished expression levels of RACGAP1 in comparison to cluster B, aligning with the notion that elevated expression of RACGAP1 correlates with a poorer prognosis (**Figure 7 E**). Additionally, it was observed that the favorable prognosis subtype within cluster A predominates in clinical staging parameters (Tumor size, Lymph Node involvement, Metastasis) (**Figure 7 F**). These findings collectively provide insights into the prognostic significance of RACGAP1 expression levels and their association with cell cycle dynamics in cancer.

### Construction and identification of prognostic models

**Figure 8:**
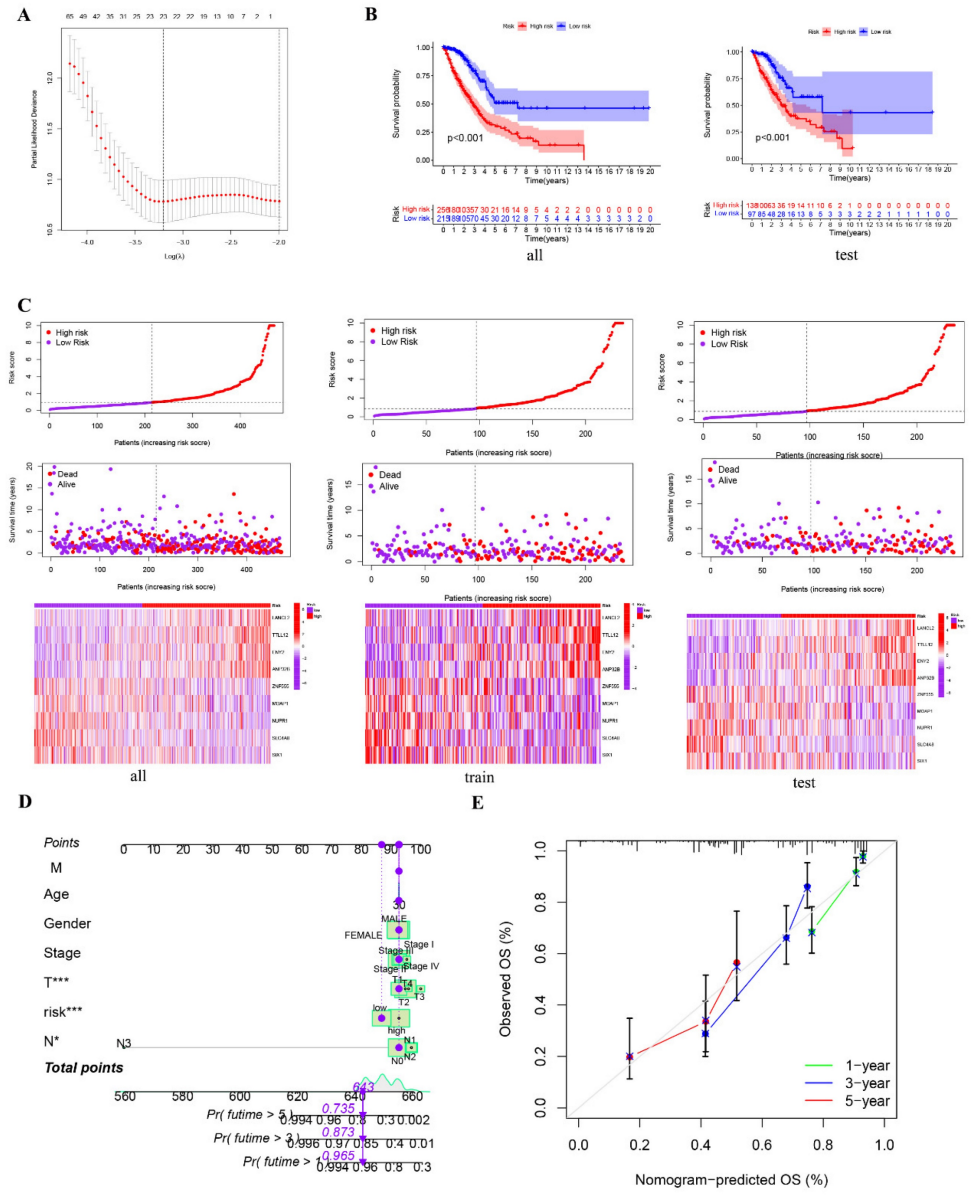
** Construction and identification of prognostic models. A** The results of LASSO regression for RrCCGs. **B** Kaplan-Meier plot for patients in a low- or high-risk group, and the number of patients in different risk groups. **C** The risk scores of LUAD patients. **D** Nomogram model **E** The calibration curve of the nomogram. (*P<0.05, **P<0.01, *** *P*<0.001).

To further substantiate the impact of RrCCGs on patient prognosis, we leveraged clinical data from TCGA cohort to successfully establish and validate a prognostic model using advanced machine learning algorithms. Employing LASSO regression and stepwise Cox regression techniques, we developed a prognostic model centered on RrCCGs, followed by the calculation of immune risk scores (**Figure 8A and additional file 2**). Stratification of patients into high and low-risk groups based on median risk scores was carried out (**Figure 8 B and Supplementary figure 3).** Survival analysis revealed substantial disparities in survival time between high and low-risk patient cohorts, with survival time progressively diminishing as risk scores escalated, as demonstrated in** Figure 8 C.** To refine prognostic predictions tailored for individual patients, we integrated conventional clinical parameters into the model, culminating in the development of a nomogram (**Figure 8 D**). Calibration curves were then employed to validate the model's predictive efficacy, affirming its exceptional prognostic value (**Figure 8 E**). These collective efforts underscore the robustness and clinical utility of the constructed prognostic model in predicting patient outcomes.

## Discussion

In this study, we systematically analyzed the role of RACGAP1 in LUAD. Firstly, we conducted a pan-cancer analysis of RACGAP1 and found it highly expressed in various cancers. Subsequently, we utilized the CancerSEA database to analyze the relevant tumorigenic cellular functions and validated them through experimental assays. Furthermore, to better serve as a prognostic reference for clinicians, we constructed a prognostic model and successfully validated it.

Through single-cell RNA sequencing data analysis, we identified associations of RACGAP1 with fundamental tumor-related cellular events, including cell cycle, DNA repair, EMT, and proliferation of stem-like cells. Before the availability of single-cell data, gene expression profiles of tumor samples could only be generated through bulk sequencing methods, potentially masking some information from stromal and immune cells. In our current study, our findings are broadly consistent with previous research. For instance, Conti et al. demonstrated that inhibiting RACGAP1 could reduce liver cancer metastasis by attenuating EMT 14. Additionally, in HCC, lncRNAs have been shown to suppress tumorigenesis by promoting demethylation of the RACGPA1 promoter, possibly mediated through the Hippo pathway 15. Similarly, RACGAP1 has been shown to inhibit the proliferation and metastasis of bladder cancer via the RACGAP1-STAT3-ESR1 pathway 16. RACGAP1 has also been demonstrated to have clinical-pathological significance in breast cancer 17.

RACGAP1, identified as a novel tumor marker gene, has been demonstrated to play a significant role in various cancer tissues. However, its involvement in lung cancer has been underreported. Employing initial analyses through CanerSEA, we investigated the fundamental oncological processes governed by RACGAP1. Our findings indicate that RACGAP1 regulates the cell cycle in LUAD cells, a discovery validated through experimental assays that aligns with prior research 18. Intriguingly, during our experimental procedures, we observed that the knockdown of RACGAP1 suppressed apoptosis, a conclusion not corroborated by database findings. Previous literature has suggested that overexpression of RACGAP can enhance renal tubular cell proliferation and inhibit apoptosis to ameliorate acute kidney injury 19. These observations contradict our findings. However, database analysis revealed that RACGAP1 affects the process of DNA damage and repair. We hypothesize that the extent of DNA damage in tumor cells surpasses the repair process, thereby promoting apoptosis. Conversely, in normal renal tubular epithelial cells, the promotion of DNA repair processes by RACGAP1 outweighs DNA damage, thus inhibiting apoptosis. Additionally, we validated that downregulation of RACGAP1 inhibits cellular invasion, metastasis, and the epithelial-mesenchymal transition (EMT) process. These findings shed light on the multifaceted role of RACGAP1 in cancer progression and underscore its potential as a therapeutic target.

Given the significant association between RACGAP1 and the cell cycle, combining RACGAP1 with its regulatory role in the cell cycle may provide more accurate prognostic indicators for tumors compared to single genes alone. We identified nine risk features of RrCCGs (RACGAP1-regulated cell cycle genes), demonstrating high accuracy in predicting LUAD prognosis. Previous research has explored several attempts to construct prognostic features using cell cycle progression genes 20-22. Prognostic features derived from cell cycle genes, called Cell Cycle Progression (CCP) scores, have been developed and validated in prostate cancer 23, 24. CCP genes have also been validated in early-stage LUAD. These findings underscore the reliability of using cell cycle genes to predict cancer prognosis 25, 26. Our study similarly constructed relevant prognostic models and validated their reliability, offering potential guidance for clinical management.

However, our study still has some limitations. While we have confirmed the influence of RACGAP1 on various biological processes of tumor cells, we cannot to fully elucidate its specific signaling pathways and targets due to experimental constraints.

## Conclusion

In summary, RACGAP emerges as a pivotal oncogene. Furthermore, it influences various processes in tumor cells, including cell cycle progression, apoptosis, invasion, and EMT. Stratification of LUAD cohorts based on RACGAP-associated cell cycle genes revealed two distinct groups with significant differences in survival and TNM staging, enabling the successful construction of a prognostic model. These findings suggest RACGAP as a potential therapeutic target and a predictive factor for survival and immunotherapy outcomes in cancer treatment.

## Supplementary Material

Supplementary figures.

Supplementary data.
