# Supplementary figures and images for: Elevated RACGAP1 Expression Enhances Malignant Potential in Lung Adenocarcinoma and Serves as a Prognostic Factor
Source: J Cancer. 2024 Jun 3;15(13):4244–58. doi: 10.7150/jca.96334 (PMC11212091; doi:10.7150/jca.96334)

++

### Protein expression of RACGAP1 in Hepatocellular carcinoma

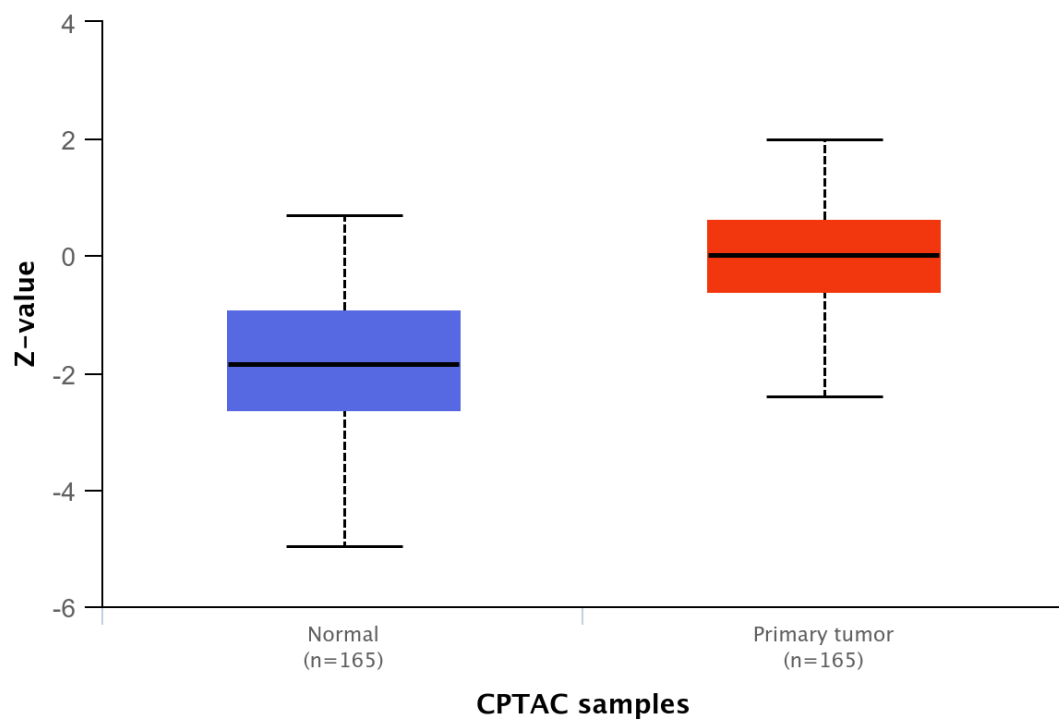

Figure 1

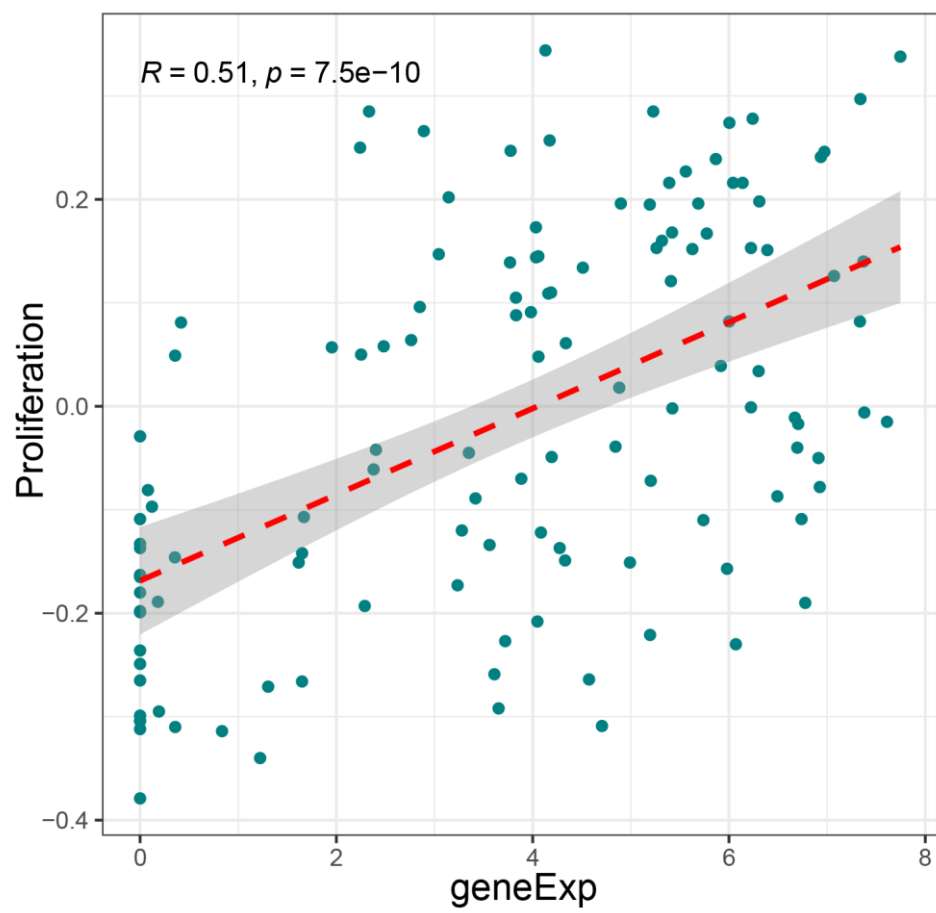

Figure 2

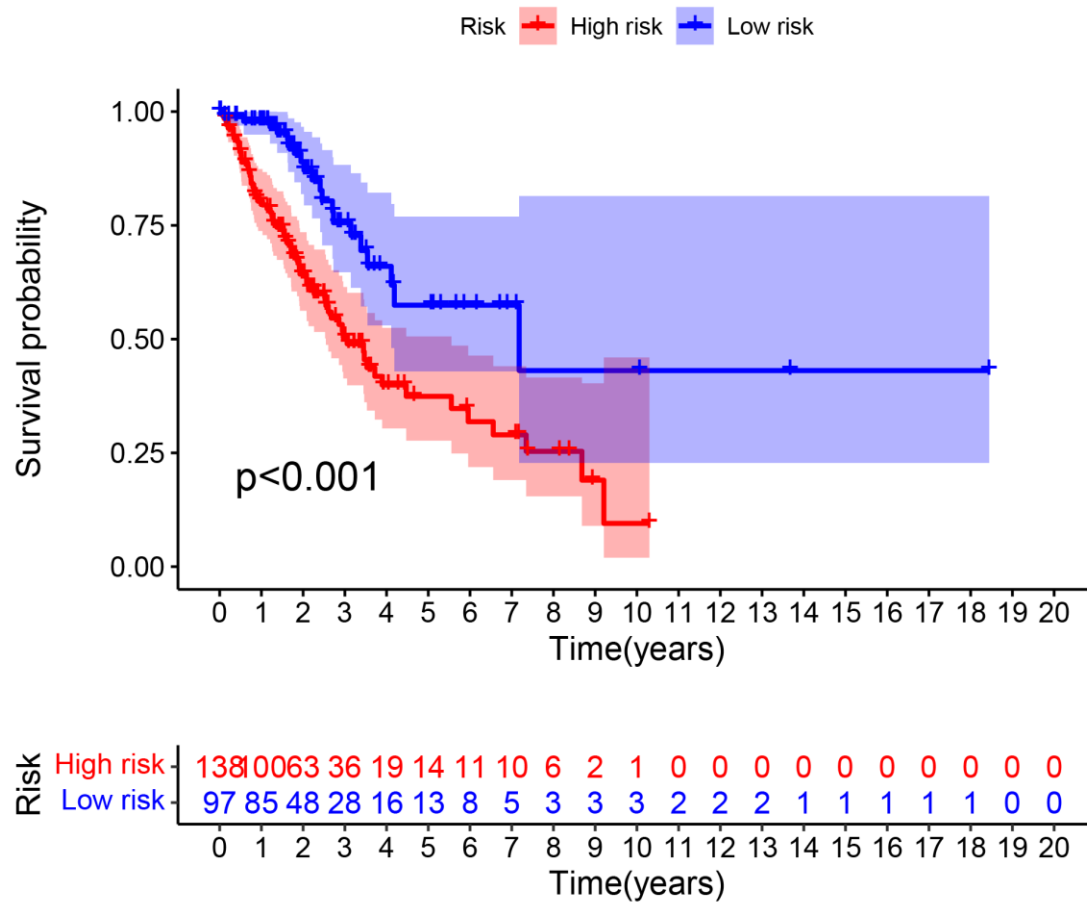

Figure 3

Supplement: Supplementary file 1 — Supplementary figures. [file jcav15p4244s1.pdf]
